# Supplementary material for: The Evolution and Characterization of the RNA Interference Pathways in Lophotrochozoa
Source: Genome Biol Evol. 2024 May 7;16(5):evae098. doi: 10.1093/gbe/evae098 (PMC11114477; doi:10.1093/gbe/evae098)
Supplement: evae098_Supplementary_Data [file evae098_supplementary_data.zip › Supplementary_materials.zip!/selected_miRNAs/CrGi_selected_miRNAs/CrGi_NC_047560.1_9304.pdf]

Provisional ID	:	NC_047560.1_9304
Score total	:	391.7
Score for star read(s)	:	3.9
Score for read counts	:	384
Score for mfe	:	2.8
Score for randfold	:	1.6
Score for cons. seed	:	-0.6
Total read count	:	765
Mature read count	:	747
Loop read count	:	0
Star read count	:	18

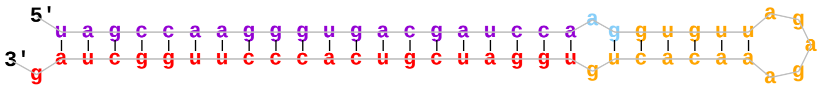

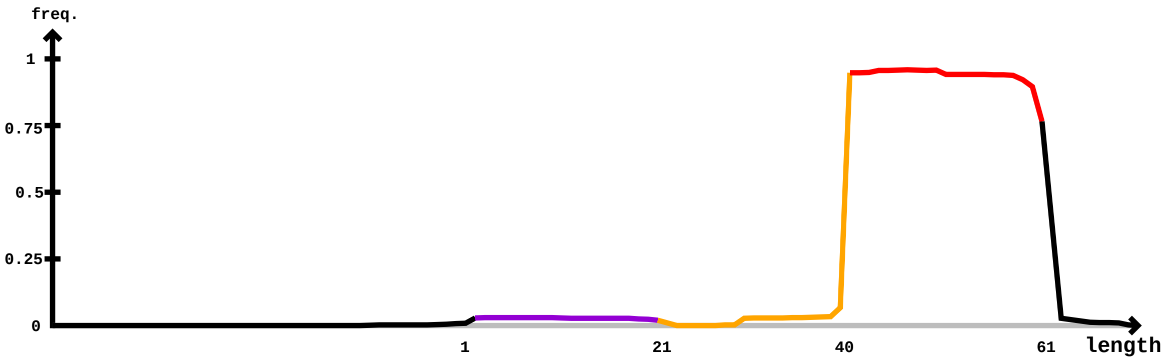

Star Mature

```
5'- cuauacagcacugaccugcuacaggucuauacccaucaucgcuagccaagggugacgauccaagguguuagagaaacacuguggaucgucacccuuggcuagcgaagaugg   -3'   obs
    cuauacagcacugaccugcuacaggucuauacccaucaucgcuagccaagggugacgauccaagguguuagagaaacacuguggaucgucacccuuggcuagcgaagaugg         exp
    ............((((((...)))))).....(((((.((((((((((((((((((((((((.((((((.....)))))).)))))))))))))))))))))))).)))))   reads   mm   sample
    ......................................ucgcuagccaagggugacgau....................................................   1       0    Mt2
    ..........................................uagccaagggugacgaucca.................................................   5       0    Mt2
    ......................................................................gagaaacacuguggaucguc.....................   1       0    Mt2
    ......................................................................gagaaacacuguggaucguca....................   2       0    Mt2
    ......................................................................gaCaaacacuguggaucguca....................   1       1    Mt2
    .............................................................................acuguggaucgucacccuCgg.............   1       1    Mt2
    ...............................................................................Aguggaucgucacccuuggcu...........   1       1    Mt2
    ................................................................................guggaucgucacccuuggc............   1       0    Mt2
    ................................................................................guggaucAucacccuuggcu...........   1       1    Mt2
    ................................................................................guggaucgucacccuuggcua..........   2       0    Mt2
    ................................................................................guggaucAucacccuuggcuag.........   1       1    Mt2
    .................................................................................uggaucgucacccuuggc............   5       0    Mt2
    .................................................................................uggaucgucacccuuggcu...........   6       0    Mt2
    .................................................................................uggaucgucacccuuggcua..........   23      0    Mt2
    .................................................................................uggCucgucacccuuggcuag.........   2       1    Mt2
    .................................................................................uggaucgucacccuuggcuag.........   181     0    Mt2
    .................................................................................uAgaucgucacccuuggcuag.........   1       1    Mt2
    .................................................................................uggaucgucacccuuggUuag.........   1       1    Mt2
    .................................................................................uggaucgucacccuuggcuGg.........   2       1    Mt2
    ....................................................................................aucgucacccuuggcuagcg.......   1       0    Mt2
    ....................................................................................aucgucacccuuggcuagcga......   1       0    Mt2
    ..........................................................................................acccuuggcuagcgaagUu..   1       1    Mt2
    ..........................................................................................acccuuggcuagcgaagaug.   1       0    Mt2

    ...............................cccaucUucgcuagccaaggg...........................................................   1       1    Mt1
    .........................................cuagccaagggugacgaucUa.................................................   1       1    Mt1
    ..........................................uagccaagggugacgaucc..................................................   2       0    Mt1
    ..........................................uagccaagggugacgaucca.................................................   3       0    Mt1
    ......................................................................gagaaacacuguggaucguc.....................   2       0    Mt1
    ......................................................................gagaaacacuguggaucguca....................   2       0    Mt1
    ......................................................................gagaGacacuguggaucguca....................   1       1    Mt1
    ..............................................................................cuguggaucgucacccuCggc............   1       1    Mt1
    ................................................................................guggaucgucacccuugg.............   1       0    Mt1
```

Star Mature

```
cuauacagcacugaccugcuacaggucuauacccaucaucgcuagccaagggugacgauccaagguguuagagaaacacuguggaucgucacccuuggcuagcgaagaugg
................................................................................guggaucgucacccuuggcu...........   1    0   Mt1
................................................................................guggaucAucacccuuggcu...........   1    1   Mt1
................................................................................guggaucgucacccuuggcua..........   3    0   Mt1
................................................................................guggaucAucacccuuggcuag.........   1    1   Mt1
.................................................................................uggaucgucacccuuggc............   1    0   Mt1
.................................................................................uggaucgucacccuuggcu...........   1    0   Mt1
.................................................................................uggaucgucacccuuggGua..........   1    1   Mt1
.................................................................................uggaucgucacccuuggcua..........   30   0   Mt1
.................................................................................uggaucgucacccuuggcuag.........   176  0   Mt1
.................................................................................uggaucUucacccuuggcuag.........   1    1   Mt1
.................................................................................uggaucgucacccuuggcuGg.........   4    1   Mt1
.................................................................................uggaucgucacccuuggcuagc........   4    0   Mt1
....................................................................................aucgucacccuuggcuagcg.......   2    0   Mt1
..........................................................................................accAuuggcuagcgaagaug.   1    1   Mt1
..........................................................................................aUccuuggcuagcgaagaugg   1    1   Mt1

.......................................cgcuagccaagggugacgaucca.................................................   1    0   Ov1
........................................gcuagccaagggugacgau....................................................   1    0   Ov1
..........................................uagccaagggugacgaucca.................................................   3    0   Ov1
....................................................................uagaCaaacacuguggaucg.......................   1    1   Ov1
......................................................................gagaGacacuguggaucguca....................   1    1   Ov1
......................................................................gagaaacacuguggaucguca....................   2    0   Ov1
......................................................................gagaaacacuguggaucgucac...................   1    0   Ov1
................................................................................guggaucgucacccuuggcu...........   2    0   Ov1
................................................................................guggaucgucacccuuggcua..........   5    0   Ov1
................................................................................guggaucgucacccuuggcuag.........   2    0   Ov1
................................................................................guggaucAucacccuuggcuagcga......   2    1   Ov1
.................................................................................uggaucgucacccuuggc............   3    0   Ov1
.................................................................................uggaucgucacccuuggcu...........   4    0   Ov1
.................................................................................uggaucgucacccuuggcua..........   23   0   Ov1
.................................................................................Aggaucgucacccuuggcuag.........   1    1   Ov1
.................................................................................uggaucgucacccuuggcuGg.........   2    1   Ov1
.................................................................................Cggaucgucacccuuggcuag.........   1    1   Ov1
.................................................................................uggaucgucacccuuggcuag.........   99   0   Ov1
....................................................................................aucgucacccuuggcuagcgaa.....   1    0   Ov1
..........................................................................................acccuuggcuagcgaagaug.   1    0   Ov1
............................................................................................cUuuggcuagcgaagaugg   1    1   Ov1

................................ccaucCucgcuagccaagg............................................................   1    1   Ov2
........................................gcuagccaagggugacgauc...................................................   1    0   Ov2
..........................................uagccaGgggugacgaucc..................................................   1    1   Ov2
..........................................uagccaagggugacgaucca.................................................   2    0   Ov2
...........................................agccaagggugacgauccaU................................................   1    1   Ov2
....................................................................uagaCaaacacuguggaucgu......................   1    1   Ov2
......................................................................gagaGacacuguggaucguc.....................   1    1   Ov2
......................................................................gagaaacacuguggaucguca....................   3    0   Ov2
......................................................................gagaGacacuguggaucguca....................   2    1   Ov2
......................................................................gagaaacacuguggauUguca....................   1    1   Ov2
.......................................................................agaGacacuguggaucguca....................   1    1   Ov2
...........................................................................acacuguggaucgucacccuC...............   1    1   Ov2
................................................................................guggaucgucacccuuggcua..........   1    0   Ov2
................................................................................guggaucAucacccuuggcuag.........   1    1   Ov2
................................................................................guggaucAucacccuuggcuagcga......   1    1   Ov2
................................................................................guggaucgucacccuuggcuagcgaa.....   1    0   Ov2
.................................................................................uggaucgucacccuuggc............   2    0   Ov2
.................................................................................uggaucgucacccuuggcu...........   4    0   Ov2
.................................................................................uggaucgucacccuuggcua..........   17   0   Ov2
.................................................................................Aggaucgucacccuuggcuag.........   1    1   Ov2
.................................................................................uggaucgucGcccuuggcuag.........   1    1   Ov2
.................................................................................uggaucgucacccuuggcuGg.........   3    1   Ov2
.................................................................................uggaucgucacccuuggcuag.........   105  0   Ov2
.................................................................................uggaucgucaccAuuggcuag.........   1    1   Ov2
.................................................................................uggaucguUacccuuggcuag.........   1    1   Ov2
.................................................................................uggaucgucacccuuggcuagc........   1    0   Ov2
.................................................................................Gggaucgucacccuuggcuagc........   1    1   Ov2
.................................................................................uggaucgucacccuuggcuagcU.......   1    1   Ov2
...................................................................................gaucgucacccuuggcuagc........   1    0   Ov2
....................................................................................aucgucacccuuggcuagcgaa.....   1    0   Ov2
......................................................................................cgucacccAuggcuagcgaag....   1    1   Ov2
.......................................................................................gucacccuCggcuagcgaa.....   1    1   Ov2
```

Star **Mature**

```
cuauacagcacugaccugcuacaggucuauacccaucaucgcuagccaagggugacgauccaagguguuagagaaacacuguggaucgucacccuuggcuagcgaagaugg
...........................................................................................Uccuuggcuagcgaagaug.    1    1    Ov2
...........................................................................................cccuuggcCagcgaagaug.    2    1    Ov2
```
